# Dengue Virus and Vaccines: How Can DNA Immunization Contribute to This Challenge?

**DOI:** 10.3389/fmedt.2021.640964

**Published:** 2021-04-12

**Authors:** Ada Maria Barcelos Alves, Simone Morais Costa, Paolla Beatriz Almeida Pinto

**Affiliations:** Laboratory of Biotechnology and Physiology of Viral Infections, Instituto Oswaldo Cruz (IOC), Fundação Oswaldo Cruz (Fiocruz), Rio de Janeiro, Brazil

**Keywords:** dengue virus, DNA vaccines, prime-boost immunization, dengue envelope protein, dengue non-structural proteins

## Abstract

Dengue infections still have a tremendous impact on public health systems in most countries in tropical and subtropical regions. The disease is systemic and dynamic with broad range of manifestations, varying from mild symptoms to severe dengue (Dengue Hemorrhagic Fever and Dengue Shock Syndrome). The only licensed tetravalent dengue vaccine, Dengvaxia, is a chimeric yellow fever virus with prM and E genes from the different dengue serotypes. However, recent results indicated that seronegative individuals became more susceptible to develop severe dengue when infected after vaccination, and now WHO recommends vaccination only to dengue seropositive people. One possibility to explain these data is the lack of robust T-cell responses and antibody-dependent enhancement of virus replication in vaccinated people. On the other hand, DNA vaccines are excellent inducers of T-cell responses in experimental animals and it can also elicit antibody production. Clinical trials with DNA vaccines have improved and shown promising results regarding the use of this approach for human vaccination. Therefore, in this paper we review preclinical and clinical tests with DNA vaccines against the dengue virus. Most of the studies are based on the E protein since this antigen is the main target for neutralizing antibody production. Yet, there are other reports with DNA vaccines based on non-structural dengue proteins with protective results, as well. Combining structural and non-structural genes may be a solution for inducing immune responses aging in different infection moments. Furthermore, DNA immunizations are also a very good approach in combining strategies for vaccines against dengue, in heterologous prime/boost regimen or even administering different vaccines at the same time, in order to induce efficient humoral and cellular immune responses.

## Introduction

Dengue is the most important mosquito-borne viral disease in the world, caused by the dengue virus (DENV). Half of the global population lives in areas at risk of infection in more than 100 countries, in tropical and subtropical regions. It is estimated that 390 million infections occur annually, in which about 96 million people develop the disease and more than 20,000 individuals die (1). The virus belongs to the Flaviviridae family, genus Flavivirus, and consists of four antigenically different serotypes (DENV1–4).

In general, dengue infections pass with minimal or no clinical signs. The disease is systemic and dynamic with a broad range of manifestations, varying from mild symptoms, the self-limiting Dengue Fever (DF), to severe dengue, the Dengue Hemorrhagic Fever (DHF), and Dengue Shock Syndrome (DSS). The DF is characterized by acute fever, often accompanied by myalgia, arthralgia, headache and rash. At defervescence time, the disease may evolve to DHF with significant plasma leakage, which can result in hemoconcentration, pleural effusion, ascites, and shock. Due to difficulties in characterizing the disease only as DF or DHF/DSS, and attempting to help in decisions of intensive monitoring and hospitalization during dengue epidemics, a new classification was established by WHO: Dengue without Warning Signs, Dengue with Warning Signs and Severe Dengue (2). Warning signs include abdominal pain, persistent vomiting, high levels of serum hepatic enzymes, hepatomegaly, exacerbated thrombocytopenia, and fluid accumulation in the lungs.

The highest difficulty for development of a dengue vaccine is to achieve protection against the four serotypes. One dengue vaccine was recently licensed (3), but phase 4 studies revealed that vaccination of DENV seronegative individuals increases DHF propensity in subsequent infections (4, 5). Thus, the development of a safe and efficient vaccine against dengue is still a priority. DNA vaccine may be an attractive strategy in this field since it can induce both arms of the immune system, with antibody production and T-cell responses. In the present paper, we provide an overview of the dengue DNA vaccines and discuss their use as single immunizations with one or more genes and heterologous prime/boost approaches. Our aim is to address the main points and strategies already studied that can contribute by improving immunogenicity and strength of DNA vaccine prototypes.

## The Dengue Virus and Infection

The DENV is transmitted to humans by female mosquitoes of genus Aedes during the blood meal, mainly *Ae. aegypti* and *Ae. Albopictus*. It first infects dendritic cells and then monocytes/macrophages, where it replicates and spreads infective particles that can reach different organs. Studies with patients that succumb to infection revealed that the virus can replicate in several organs, such as the liver, heart, lung, and brain (6–8). It interacts with different cell receptors through the E protein [reviewed in (9)].

**Figure 1:**
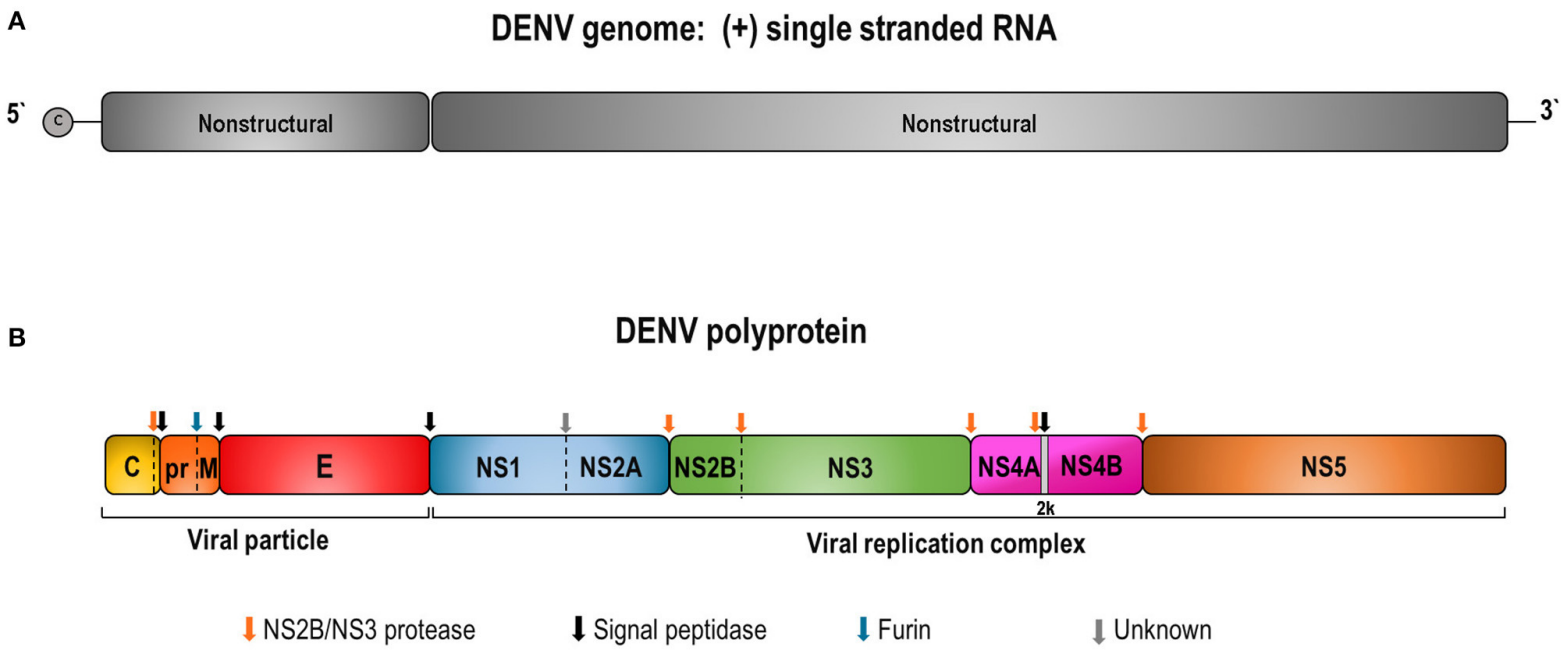
Schematic representation of DENV genome **(A)** and the polyprotein precursor of viral proteins **(B). (A)** DENV contains a capped single-stranded and positive-sense RNA genome of about 10,700 bases in length. The DENV genome is an mRNA with one open reading frame encoding a single precursor polyprotein of about 3,400 amino acids. **(B)** The polyprotein cleavage by the viral NS2B/NS3 and host cell proteases generates 3 structural (C, prM/M, and E) and 7 non-structural (NS1, NS2A, NS2B, NS3, NS4A, NS4B, and NS5) proteins. Colored arrows indicate enzyme cleavage sites by the different proteases.

Dengue genome is a single-stranded RNA, positive-sense, with ~11 Kb. It encodes a polyprotein precursor that is processed by viral and host cell proteases to produce three structural (capsid—C; pre-membrane/membrane—prM/M; envelope—E) and seven non-structural proteins (NS1, NS2A, NS2B, NS3, NS4A, NS4B, and NS5) (Figure 1) (10).

The transmembrane E glycoprotein is the major component of the virion surface and it is associated with numerous biological activities. This protein is organized in dimers, each monomer composed of three domains (I, II, and III), and domain III is responsible for virus-host cell recognition. After interaction and virus internalization in vesicles, pH reduction occurs in the phagolysosomal compartment, turning the dimers into trimers and exposing its fusion peptide present in domain II, which mediates fusion of virus and host cell membranes (10, 11). The viral genomic RNA is then released and the polyprotein is translated in the endoplasmic reticulum (ER). Since the first contact of DENV and host cells is mediated by E protein, this is the main target for eliciting neutralizing antibodies (NAb). Hence, most vaccine strategies are based on this protein (12).

The prM protein, in turn, acts as a chaperone that maintains correct conformation of E protein during virus assembly. The prM is cleaved by furin in the trans-Golgi, resulting in mature virions that contain the nucleocapsid surrounded by a host-cell-derived membrane, in which the E and M proteins are anchored (9, 13). This protein is also targeted for the development of NAb (12). However, other studies demonstrated that anti-prM antibodies are highly cross-reactive among the different DENV serotypes and may be involved in the dengue pathogenesis (14, 15).

The C protein has a hydrophobic portion that interacts with the ER membrane and another basic region that binds to the viral RNA to form the nucleocapsid, which encompasses the genomic RNA in the new virus particles. Apart from the genome encapsidation, the C protein seems to have additional roles. It can leave replication compartments and enter the nucleus, which suggests its involvement in host transcriptome changes (9, 16), and may participate in apoptosis and cell cycle arrest mechanisms (17).

Non-structural proteins are involved in viral polyprotein processing, replication, and innate immune antagonism. The glycoprotein NS1 can be found within infected cells as monomers, associated with the surface of these cells as dimers or secreted as hexamers (18, 19). During the acute phase of the disease, NS1 circulates in large quantities in blood patients, making it an important tool for dengue diagnosis as an early infection marker (18, 20). This protein colocalizes with double-stranded RNA in the lumen of the ER in infected cells, suggesting its role in viral replication (21, 22). It also interacts with host proteins, as well as with the NS4A and NS4B, probably assisting the anchoring of the viral replication complex to membranes (23, 24). Moreover, recent studies with a proteomic approach reveal that expression of the dengue NS1 in hepatic cells results in up and downregulation of several host proteins (25). Some investigations suggest its role in hemorrhage and vascular leakage by biding to prothrombin, with interruption of the coagulation cascade (26). Besides, anti-NS1 antibodies were reported to cross-react with host platelets, interfering with its aggregation, and to endothelial cells causing damages (24, 27). However, most of these studies were performed with antibodies generated by non-glycosylated NS1 expressed in bacteria and, therefore, with different conformations compared to the native protein, which may have impacted the observed results. On the other hand, other studies emphasize the NS1 potential as a protective antigen that can be used in vaccine development.

The NS2A is also part of the replicative complex and it helps inhibition of host interferon α/β signaling pathways mediated by NS4A and NS4B proteins (28). The NS2B, in turn, acts as a cofactor for the NS3, which is responsible for cleavage of the viral polyprotein in specific points (29). The NS3 is a multifunctional protein involved in polyprotein processing, RNA capping, and RNA replication. It has a serine protease domain at the N-terminal region and helicase, RNA triphosphatase, and nucleotide triphosphatase activities at the C-terminal portion (30, 31). The NS4A participates in the rearrangement of intracellular membranes, essential for viral replication. It can also induce autophagy, which increases infection (32, 33). NS4B is capable of modulating viral replication through its interaction with the helicase domain of the NS3 protein (34).

The NS5 is the largest non-structural protein encoded by the DENV genome and is highly conserved. Its N-terminal portion has a methyltransferase domain responsible for capping and methylating the 5′ end of the viral RNA, and the C-terminal region acts as RNA-dependent RNA polymerase. Similar to NS4A and NS4B, NS5 can also interfere with the host IFN-α/β pathway (35). For DENV genome replication, copies of single-stranded RNA negative-sense are made by the NS5. These intermediate RNA serve as the template for the synthesis of several other positive-sense RNA molecules that are incorporated into new viral particles.

## The Pathogenesis of Dengue and Immune Responses

Infection with one serotype of dengue induces long-term protection against this serotype, but only temporary protection against other DENVs. Also, and more important, secondary infection with a heterologous serotype increases the chance of developing severe dengue (36, 37). It seems that immunity to the first DENV leads to exacerbated and non-protective immune responses against the second serotype.

A characteristic of dengue, especially in the DHF, is the increase of pro-inflammatory and vasoactive cytokine levels in the plasma (38). The excessive immune activation leads to a cascade of cytokine production, named as cytokine storm, that increases vascular permeability and correlates with disease severity. High serum levels of IFN-γ, TNF-α, IL-1β, IL-4, IL-6, IL-7, IL-8, IL-10, IL-13, IL-15, IL-17, IL-18, macrophage migration inhibitory factor (MIF), CL2, CCL4, CCL5, and CXCL10 (IP-10) have already been reported in patients with DHF (39, 40).

Humoral and cellular immune responses are activated during DENV infections and may play critical roles in viral clearance as well as in the disease pathogenesis. Induction of neutralizing antibodies constitutes the first line of defense against future infections with the same viral serotype. However, one hypothesis to explain the development of severe dengue after a second infection with a heterologous serotype is the antibody-dependent enhancement of virus replication (ADE). In this case, anti-E and mainly anti-prM antibodies generated by the first infection bind to the second serotype without neutralizing efficacy. Instead, these antibodies may augment heterotypic virus entry in primary target cells such as macrophage and dendritic cells, by interacting with Fc receptors, resulting in enhancement of viremia and the cytokine storm (41–43). Thus, ADE seems to be the major obstacle for dengue vaccine development (44).

Another hypothesis, named original antigenic sin and not necessarily exclusionary to the ADE, is based on the cellular immune response. Thereby, cross-reactive memory T cells generated by the first infection are preferentially activated during the second infection with another virus serotype. These cells would have low-affinity for antigens of the second DENV, would be unable to clear infection and could contribute to cytokine storm which predisposes plasma leakage and severe disease (45). Recently, Talarico and coworkers reported that heterologous CD8^+^ cells may induce pathology after infection in immunocompetent mice (46).

However, the role of the T-cell response in dengue infection is far from being completely understood. Studies with healthy individuals from dengue-endemic regions revealed, for instance, high T-cell recognition directed to epitopes that are conserved among all DENVs. Furthermore, concerning the magnitude, avidity, or cytokine production, T-cell responses toward these epitopes were not different from those directed to serotype-specific ones (47). Other studies have shown temporal mismatch between CD8^+^ T-cell response and the arise of capillary leakage, which contradicts the idea that these cells are responsible for early triggering of hemorrhage in children with DHF (48). Additionally, recent investigations suggest that HLA alleles related to increased risk of severe dengue are also associated with weak CD8^+^ T cell responses (49). CD4^+^ T lymphocytes, including cytotoxic CD4^+^ T cells, in turn, seem to play important role in protection against severe dengue development (50). Moreover, analysis of different cytokine production by DENV-specific T cells revealed the importance of multifunctional T-cell responses in protection against dengue infection (51, 52).

All these results have a direct impact on vaccine development. Indeed, nowadays it is almost unanimous that a dengue vaccine should induce both neutralizing antibodies and protective T-cell response. However, almost all vaccine strategies against dengue that have been developed for decades are based primarily on the induction of neutralizing antibodies.

## Dengue Vaccines

It is a consensus that a dengue vaccine must be effective against the four virus serotypes, to avoid the risk of severe dengue development in patients infected with one serotype not included in vaccination. The only licensed dengue vaccine is the Dengvaxia, produced by Sanofi Pasteur and registered in several endemic countries, in Europe, and in the US. This vaccine is composed of four live-attenuated chimeric viruses with the backbone of yellow fever virus, another flavivirus with a genomic organization similar to dengue, in which prM and E genes were substituted by these genes from the four DENV (53). Results from phase 2 clinical trials with children in Asia pointed out that this vaccine was not fully protective against all serotypes, especially against DENV2 (3). These studies indicated that induction of neutralizing antibodies may not be sufficient to provide protection, since those children did present significant levels of NAb against all dengue serotypes, and suggested the importance of T-cell responses. The report also pointed out that other DENV antigens not present in Dengvaxia, such as non-structural proteins, may be important for generation of robust protection. Phase 4 studies with vaccinated individuals in the Philippines and Brazil revealed that DENV seronegative people, mainly children, became more susceptible to develop DHF when infected after vaccination (4, 5). Such observations led WHO to recommend vaccination with Dengvaxia only to dengue seropositive individuals. Nevertheless, Dengvaxia is considered efficient for protection in seropositive individuals for at least 5 years (54).

Several other vaccines have been investigated in preclinical studies using different animal models and some have moved on to clinical trials (55, 56). Currently, there are two dengue vaccines in phase 3 trials based on live-attenuated viruses (TV003 and TAK-003). The TV003 is composed of a viral mixture in which 30 nucleotides in the 3′ UTR of DENV1, DENV3, and DENV4 were deleted for attenuation, and prM/E genes from attenuated DENV4 were substituted by the same DENV2 genes, generating DENV2/4 (57). The vaccine was developed in the US (NIH) and is been tested in Brazil by the Instituto Butantan (Butantan-DV). Results from phase 2 clinical trials with DENV-naïve and DENV-exposed healthy volunteers showed seroconversion between 76 and 92% against the different serotypes (58). Vaccinated individuals also presented CD8^+^ T-cell responses to non-structural dengue proteins with IFN-γ production. However, neutralizing antibody titers were 2–4 times higher in DENV-exposed individuals compared to DENV-naive participants. Moreover, data revealed an association between antibody response and the appearance of adverse reactions, especially rash.

The TAK-003, produced by Takeda, is based on the backbone of a DENV2 attenuated strain obtained by serial passages in cell culture and chimeric virus generated by the replacement of the DENV2 E and prM genes by those of other serotypes (59). The vaccine is being evaluated in a multi-country phase 3 trial in Asia and Latin America, in children and adolescents. After 18 months post-vaccination, analysis revealed the efficacy of 76 and 66% in DENV-exposed and -naïve individuals, respectively (60). Efficacy varied depending on the virus serotype, with high levels for DENV2 and DENV1 and less efficiency to DENV3 and DENV4. Despite the relatively low efficacy of this vaccine, the study suggested a reduction in the number of hospitalizations with severe dengue cases. Besides, the high protection rate against DENV2 may be a consequence of a cellular immune response toward DENV2 non-structural proteins together with NAb.

One important issue regarding attenuated virus-based vaccines is the competition between these viruses, which may result in imbalanced immune responses. In contrast, inactivated tetravalent vaccines could induce more equilibrated immune responses toward the four serotypes. A tetravalent dengue purified inactivated vaccine (DPIV) was evaluated in phase 1 clinical trials in the US and Puerto Rico, in study designs developed collaboratively with the Walter Reed Army Institute of Research and GlaxoSmithKline (GSK) (61, 62). However, development of inactivated dengue vaccines is hampered by their low immunogenicity, mostly by the lack of robust T-cell responses, and possibly by the absence of responses against DENV non-structural proteins.

Subunit vaccines could also be an option to overcome the problem of unbalanced immune responses against different DENVs. One dengue subunit vaccine is based on truncated E protein (DEN-80E), constituting its ectodomain, expressed in Drosophila S2 cells (63). The monovalent DENV1 vaccine was tested in flavivirus-naïve adults in phase 1 clinical trials, sponsored by Hawaii Biotech (64) and a tetravalent formulation was further evaluated by M&Co (65). The vaccine was tested with different adjuvants, since administration only with purified proteins tends to induce low immune responses. One formulation showed robust NAb response and even IFN-γ production, but it was also associated with more adverse events.

Another strategy is the nucleic acid vaccines based on DNA or RNA. DNA vaccines consist of plasmids encoding one or more genes from a specific pathogen, which may be administrated directly into appropriate tissues. *In vivo* expression of the target antigen driven by efficient eukaryotic promoters and endogenous post-translational modifications results in native protein structures ensuring appropriate folding and immune presentation. DNA vaccines simulate a viral infection with stimulation of both B- and T-cell responses, without the risk of using an infectious agent (66). Besides, DNA vaccines have other advantages over traditional approaches, including the relatively easy development and large-scale manufacture, and stability at ambient temperatures without the need for cold chain, which may impact the final cost of its production and distribution. Another important characteristic of DNA vaccines is that they can be readily manipulated and engineered to answer questions about the importance of specific epitopes, for instance, and rapidly target emerging infectious diseases (67).

Several preclinical studies involving DNA vaccines against DENV have been published in the last few years and will be discussed below. Initial human trials with DNA vaccines in general induced poor immune responses. Early, DNA delivery was performed mainly by intramuscular simple injection, leading to low transfection efficiency in primates. Afterward, different administration approaches had been developed, including electroporation, which causes temporary fenestration of membranes and increases plasmid passage into the cells and the nuclei. Further strategies to improve the potency of DNA vaccines have been developed focusing either on increasing the amount of recombinant protein production, such as codon optimization, or addition of adjuvants and other immunomodulators, such as cytokines and co-stimulatory molecules (68, 69).

Safety concerns about DNA vaccines have been raised since initial studies, mainly regarding their possibility to integrate in the host cell genome. Wolff and coworkers demonstrated that one plasmid inoculated in mouse muscle cells remained only in a non-integrated manner 2 years after administration (70). In contrast, low level integration was observed after electroporation, although with lower frequencies compared to spontaneous gene mutations (71). Further human studies with DNA vaccines also demonstrated its safety and integration is no more a major concern (72).

So far, no human DNA vaccine is already available, however it is important to note that the first licensed DNA vaccine in the world was precisely against another flavivirus, the West Nile Virus (WNV), this vaccine, based on the prM and E proteins, is used for immunization in horses (73). It is also worth mentioning that nowadays some DNA vaccines against the severe acute respiratory syndrome coronavirus 2 (SARS-CoV2) are being tested in phase 3 clinical trials (74).

Recently, RNA vaccines have gained a lot of attention with the SARS-CoV2 pandemic and are currently being administered to the population in several countries (75). The RNA vaccines are composed of messenger RNAs generally formulated with lipids to increase their stability and allow entry into host cells. Both DNA and RNA vaccine studies initiated almost simultaneously in the 90th decade and are in general able to induce humoral and T cell responses (72). One RNA vaccine based on conserved and highly antigenic epitopes located in the DENV NS3, NS4B, and NS5 regions was tested in human transgenic mice. It induced strong T cell responses and protection after dengue challenge (76). The ease of making nucleic acid vaccines is an enormous advantage for rapid responses to emergent or epidemic diseases such as SARS-CoV2 and Zika (77, 78). However, RNA vaccines need storage at low temperatures making them more expensive compared to DNA vaccines.

## Dengue DNA Vaccines

### Vaccines Based on prM and E Proteins

Most dengue DNA vaccines are also based on the E protein as it has the largest number of epitopes targeting for neutralizing antibodies. Since during virus replication the E protein needs prM as a chaperone to achieve its final native conformation, many groups address the use of these two antigens together in the design of DNA vaccines. The DENV vaccines based on prM and E proteins that will be detailed in this review are summarized in Table 1.

**Table 1:** DNA vaccines based on dengue structural proteins.

**Vaccine (study ID)**	**Antigen (serotype)**	**Plasmid vector**	**Strategy (inoculation route)**	**Animal model**	**Immunogenicity**	**Protection**
p1012D2ME + pUC 19 (79)	prM and E (DENV2)	pVR1012	Plasmid encoding prM and 92% of E + pUC19 plasmid with CpG motifs (i.d.)	BALB/c mice	Production of anti-DENV2 Ab Positive PRNT_50_ Ab responses were higher in DNA vaccine + pUC19 group	60% protection in mice immunized with DNA vaccine plus pUC19 against an i.c. challenge with DENV2
pD2MEL (80)	prM and E (DENV2)	pVR1012 + VR1701	Plasmid encoding prM and 92% of E fused to LAMP-1 membrane anchor and cytoplasmic domains + GM-CSF plasmid (VR1701)	Mice	Production of anti-DENV2 Ab Positive PRNT_50_ Antibody responses were higher in prM/E/LAMP-DNA vaccine + VR1701 group	Not analyzed
D1ME100 Clinical Trial (81–83)	prM and E (DENV1)	pVR1012	Plasmid encoding prM and 92% of E (i.m.)	44 healthy dengue-naïve volunteers	Production of anti-DENV1 IgM and IgG Positive PRNT_50_ Positive IFN-γ-ELISPOT	Not analyzed
TVDV^VAX^ (80–82, 84–86)	prM and E (DENV1–4)	pVR1012	Plasmids encoding DENV1, 3, and 4 prM and 92% of E + plasmid encoding DENV2 prM and 92% of E fused to LAMP-1 (i.m.)	Rhesus monkeys (i.m.)	Positive PRNT50: NAb against DENV1–4 Positive IFN-γ -ELISPOT responses: DENV1 and DENV2-specific T-cell responses	Absence or lower levels of viremia in monkeys after DENV2 s.c. challenge
Tetravalent formulation TVDV^VAX^ Clinical Trial (87)	prM and E (DENV1–4)	pVR1012	Plasmids encoding DENV1, 3, and 4 prM and 92% of E + plasmid encoding DENV2 prM and 92% of E fused to LAMP-1 (i.m)	40 healthy dengue-naive adult volunteers	Negative ELISA or very low Ab titers Negative PRNT_50_	Not analyzed
pCID2Et (88)	E (DENV2)	pCI	Plasmid encoding the E signal sequence and 94% of E (i.m.)	BALB/c mice	Production of anti-DENV IgG not detected Production of anti-IFN-γ and IL-4 not detected	20% protection in mice against i.c. challenge with DENV2
pcD1ME, pcD2ME, pcD3ME, and pcD4ME (89–91)	prM and E (DENV1–4)	pcDNA3	Plasmids encoding prM and E of DENV1–4 (i.m.)	BALB/c mice	Production of anti- DENV1–4 IgGs Positive PRNT_70_: monovalent and tetravalent formulations against DENV1–4	Not analyzed
7pDIII-D1, pDIII-D2, pDIII-D3, and pDIII-D4 (92)	EDIII (DENV1–4)	pcDNA3	Plasmids encoding EDIII of DENV1–4 (i.m.)	BALB/c mice	Production of anti- DENV1–4 IgGs NAb in a 50% BHK-21 cells CPE assay: tetravalent formulation against DENV2 only	87% protection in newborn BALB/c mice inoculated intracranially with sera from tetravalent-immunized mice mixed with DENV2 NGC
pDV-U-DIII (93)	EDIII (DENV1–4)	pVAX1	Plasmid encoding EDIII of DENV1–4 as a single open reading frame (consensus human codon optimized sequence) (i.m.+e.p.)	BALB/c mice	Production of DENV1–4 anti-EDIII IgGs NAb against DENV1–4 in TCID50-CPE inhibition assay Antigen-specific antibody-producing B cells after incubation with EDIII proteins of DENV1–4	Not analyzed
pE1D2 (94–96)	E (DENV2)	pcDNA3	Plasmid encoding t-PA signal peptide and 80% of E (i.m.)	BALB/c mice	Positive PRNT_50_ assay Positive IFN-γ and TNF-α ELISPOT responses: DENV2-specific CD4^+^ and CD8^+^ T cells (immune epitope mapping)	90–100% survival and 10% morbidity rates in mice against i.c. challenge with DENV2
pE2D2 (94)	E (DENV2)	pcDNA3	Plasmid encoding t-PA signal peptide and EDIII (i.m.)	BALB/c mice	Negative PRNT_50_	45% survival and 65% morbidity rates in mice against i.c. challenge with DENV2
DIII-CH3 monovalent and tetravalent (97)	EDIII (DENV1–4)	pVAX	Plasmids encoding EDIII of DENV1–4 from codon-optimized EDIII sequences fused to γCH3 (i.d.)	BALB/c mice	Production of anti-EDIII DENV1, 2, 3 (monovalent and tetravalent vaccination) Positive PRNT_70_: monovalent and tetravalent formulations against all 4 DENV serotypes	Not analyzed
EDIII DNA vaccine (98)	EDIII (DENV1–4)	pVAC1-mcs	Plasmids encoding EDIII of DENV1–4 as a single open reading frame (i.m.)	BALB/c mice	Positive NAb against DENV1–4 in TCID_50_-CPE inhibition assay	83.3% protection in mice against DENV1, 3, and 4 i.c. challenge and 50% protection against DENV2 challenge
TetraME (99–102)	prM and E (DENV1–4)	pVAX	Plasmids encoding prM and E (i.m.+e.p.)	BALB/c mice	Production of anti-DENV1–4 IgGs Positive PRNT_50_: Nab against DENV1–4 Positive IFN-γ- and IL-2-ELISPOT responses	100% protection in mice against DENV1–4 i.c. challenge 30 weeks after immunization
scDEC-EDIII (103)	EDIII (DENV2)	pcDNA3	Plasmids encoding EDIII fused to scFv aDEC205 (i.m.+e.p.)	BALB/c mice	Positive ELISA: anti-EDIII IgG titers Positive IFN-γ, TFN-α, IL-2 ELISPOT: DENV2-specific CD4 T cells (immune epitope mapping) Positive NT50: anti-EDIII antibodies were able to block DENV2 infectivity by binding to EDIII	Not analyzed

*Some reports of monovalent DNA constructions that have resulted in further studies with tetravalent formulations have not been included in this table. References from previous work with monovalent vaccines have been included in the “Vaccine (study id)” column for more detailed information. i.c., intracerebral; i.m., intramuscular route; s.c., subcutaneous; i.m.+e.p., intramuscular + electroporation; NAb, neutralizing antibodies; PRNT, plaques reduction neutralization test; EDIII, domain III of envelope*.

The first report of prM- and E-based nucleic acid vaccine dates from the early 1990s and originated the DNA vaccine that is currently undergoing clinical trials. Kochel and colleagues developed a monovalent DNA vaccine containing the prM signal peptide sequence, localized in the C-terminal end of the capsid gene, the prM gene and 92% of the E sequence without its transmembrane portion, all from DENV2 New Guinea C (NGC) strain (104). Sequences were cloned into the pVR1012 plasmid, generating the p1012D2ME vaccine. BALB/c mice immunized intradermally with multiple doses of p1012D2ME showed specific anti-DENV2 NAb, however, vaccination failed to protect animals against a lethal intracerebral DENV2 challenge. Subsequent studies revealed antibody response improvement and a 60% survival rate in BALB/c mice co-immunized with pUC19 plasmid that contains CpG motifs (79), emphasizing the possibility of improving immunity by using these immune-stimulatory motifs.

The same group constructed a DENV1 DNA vaccine similar to p1012D2ME (p1012D1ME100), encoding prM and 92% of E protein from Western Pacific 74 strain. This vaccine elicited higher NAb titers in BALB/c mice when compared to immunization with another construction encoding only truncated E protein (83). The immunogenicity of p1012D1ME100 was tested in non-human primates (NHP) inoculated three times, by the intramuscular (i.m.) or intradermal (i.d.) route (81, 86). These studies presented the first proof of concept about the efficiency of a dengue DNA vaccine in monkeys. In Aotus monkeys, i.d. inoculation induced better antibody response compared to the i.m. route. One animal in each group (*n* = 6) became completely protected against a subcutaneous DENV1 challenge, while others presented a viremia decrease (81). In contrast, full protection was observed in four rhesus monkeys (*n* = 8) immunized by i.m. route and no protection in i.d.-inoculated animals (86). Interestingly, rhesus monkeys showed no detectable NAb at the viral challenge point, raising doughs of which immune components were indeed responsible for protection. The T-cell response was assessed by IFN-γ production but no positivity was found, probably by the use of inactivated DENV1 for lymphocyte stimulation in the *in vitro* assay, which is not as efficient as purified proteins or peptides.

To improve immunogenicity of these vaccines, authors replaced the carboxy-terminal of the DENV2 E gene by sequence coding membrane anchor and cytoplasmic domains of LAMP-1H (pD2MEL). The C-terminal sequence of the lysosome-associated membrane protein (LAMP) targets the protein to the lysosomal membrane and can improve antigen processing for presentation *via* MHC-II. Mice injected with the pD2MEL by i.m. route and presented better antibody response, which was further increased by co-immunization with a plasmid encoding the granulocyte-mouse monocyte colony-stimulating factor (GM-CSF) (80). The benefits of antigen presentation through MHC-II after DNA immunization for NAbs production were also seen in a second report of this group (105).

These results drew attention to the possibility of improving DNA vaccine platforms with immune-stimulatory mediators and encouraged similar studies in Aotus monkeys inoculated with the DENV1 vaccine (p1012D1ME100). Authors compared co-immunization with plasmids containing GM-CSF or human immunostimulatory sequences (ISS), using a needle-free system. Vaccination with this mixed formulation led to complete protection in most monkeys when challenged 1 or 6 months after immunization (106). However, the advantage of such immunostimulatory sequences is not a consensus. In fact, BALB/c mice immunized with another prM/E DNA vaccine showed a negative impact in immunogenicity and protection when co-inoculated with a GM-CSF plasmid (107).

In 2005, a similar DENV3 prM/E-derived vaccine, p012-D3ME, with sequence from Asian strain was described as immunogenic in Aotus monkeys following a 3-dose immunization schedule by i.d. route. One monkey showed no viremia by RT-PCR after DENV3 challenge, while 2 other animals presented lower viral copies (*n* = 6) (84).

Subsequently, the DENV1 vaccine (D1ME100) proved to be safe, without severe adverse events in a phase 1 clinical trial with 22 dengue-naïve volunteers (82). Only 41% of volunteers seroconverted with NAb after immunization with the highest DNA dose (three i.m. injections of 5 mg in 0-,1-, and 5-months), using a needle-free Biojector 2000, while a tetravalent T-cell response with IFN-γ production upon stimulation with E proteins from all dengue serotypes was noted after both low and high DNA doses. These results revealed the potential of DNA vaccines in inducing robust cell responses despite poor antibody production. A follow-up pre-clinical trial was conducted in NHPs immunized with a tetravalent dengue DNA vaccine (TVDV), composed of the plasmids described above (80, 81, 84) and a DENV4 vaccine encoding prM/E genes from a Philippine strain, administered with Vaxfectin adjuvant (85). Results showed increased NAb levels against DENV1, 3, and 4 compared to vaccination without Vaxfectin, but not against DENV2, and almost no change regarding the T-cell response. Animals were challenged with DENV2 and protection was confirmed by reduced viremia (85). The TVDV-Vaxfectin formulation also elicited strong antibody responses in New Zealand white rabbits toward the four serotypes (108).

The TVDV formulation was then evaluated in phase 1 clinical trial with 40 dengue-seronegative healthy volunteers i.m. immunized in a 0-/30-/90-day schedule, combined or not with Vaxfectin adjuvant (82). TVDV-Vaxfectin failed to elicit efficient NAb responses, but induced production of IFN-γ in about 80% of volunteers. Although improvement of anti-dengue neutralizing antibody responses is extremely required, there is also strong evidence that cellular immunity and specific polyfunctional T-cells are important (109). Consequently, such results encourage the development of dengue vaccines betting on heterologous prime and boost protocols, with DNA immunization contributing to the induction of robust cellular immune responses. Besides, human trials with DENV DNA immunizations revealed no genomic integration or neoplastic alterations that could be a risk concerning DNA vaccines.

Another approach is based on the commercial pcDNA3 plasmid and encodes the signal sequence of prM and prM/E genes from DENV2 NGC virus, designated as pcD2ME (89). Most BALB/c mice i.m. immunized with two pcD2ME doses showed borderline NAb titers and a third DNA dose did not increase the antibody response. Nevertheless, another similar DNA vaccine with prM/E genes from DENV1 Mochizuki strain, pcD1ME, elicited higher NAb titers in ICR mice immunized twice with a spring-powered needle-free jet injector (90). These studies culminated in a tetravalent formulation along with the newly constructers containing genes from DENV3 H87 and DENV4 H241 strains (pcD3ME and pcD4ME, respectively), using needle-free DNA inoculation system (91). Two doses of the tetravalent formulation induced similar antibody titers as the monovalent vaccines in BALB/c mice, except for pcD4ME which elicited higher antibody levels when administered in the tetravalent formulation. Long-term immunity was observed, with NAb detectable 30 weeks after the first DNA dose. Authors also showed that the immunogenicity of this tetravalent formulation can be increased when combined with a subunit vaccine (110).

Recent attempts involved improvement of DENV1 and DENV2 DNA vaccines by cloning into pVAX vector the prM/E cassettes derived from DENV1 West Pacific and DENV2 NGC strains, (pVAX1-D1ME and pVAX1-D2ME, respectively) (99, 100). Given the poor protection induced in mice immunized by the i.m. route with these vaccines (30% survival rate), they were inoculated by electroporation (e.p.). pVAX1-D2ME-immunized BALB/c mice presented more than a 10-fold increase of DENV2-specific antibody titer and a 4.5-fold increase in the NAb levels when compared to animals inoculated intramuscularly, and higher production of IFN-γ, IL-2, IL-4, and IL-10 and CTL activity (99). Survival rates reflected differences in the induced immune responses. About 90% of BALB/c mice immunized by e.p. with pVAX1-D2ME survived DENV2 intracranial challenge, while all animals i.m. inoculated succumbed to infection. Electroporation with pVAX1-D2ME vaccine also induced partial protection in a passive immunization assay with SCID mice inoculated with a serum mixture from e.p.-immunized BALB/c mice and a DENV2 suspension (99). These studies represent, therefore, a clear example of how the inoculation route can influence immunity mediated by DNA vaccines.

Following these and other reports presenting promising results with monovalent prM/E-based pVAX constructions (99–102), a new tetravalent formulation was developed by cloning sequences of Hawaii, Tr1751 strain, H87 and H241 strains from DENV1 to 4, respectively (TetraME) (78, 102). A 3-dose scheme of 50 μg of each monovalent DNA vaccine administered by electroporation in BALB/c mice induced T-cell activation with high IL-4 and IFN-γ production, long-term NAb response against all the four serotypes, and animals survived monovalent DENV1–4 challenges (78).

Other groups also developed dengue DNA vaccines based on prM/E proteins. DePaula and coworkers constructed candidates encoding prM and E proteins from DENV3 H87 strain which elicited NAb and Th1 cytokine production in BALB/c mice and generated 80% protection against DENV3 (111). Investigation of antibody levels raised in mice inoculated with prM/E vaccines based on two distinct genotypes of DENV 1 and 3 revealed higher NAb titers after immunization combining genes from homologous genotypes compared to heterologous ones (112). One DENV2 prM/E vaccine derived from Cosmopolitan genotype was evaluated in mice to understand how differences in amino acid sequences may affect cross-protection against different genotypes (113). Data revealed that induced antibodies could neutralize both Cosmopolitan genotype and NGC reference strain. These are encouraging results since several genotypes of the same dengue serotype can circulate in endemic regions and a vaccine should protect against all of them.

In general, vaccines based on prM/E proteins can produce virus-like particles (VLPs), which may induce antibodies against conformational epitopes essential for viral neutralization (114, 115). Unfortunately, information about the existence of VLPs generated by prM/E-based DNA vaccines is scarce (83, 90).

DNA shuffling strategies were used in attempting to shorten antigen sequences against all dengue serotypes in a single prM/E DNA vaccine, with codon-optimized sequences for improving expression in mammalian cells (116). Three of these vaccines induced tetravalent NAb and T-cell responses in BALB/c mice, leading to partial or complete protection after a lethal intracranial DENV2 challenge. Follow-up studies were conducted in NHPs, with two vaccines encoding prM and E proteins (sA and sC) and one containing only the envelope ectodomain sequence (sB) (117). Only prM/E-derived shuffled vaccines induced tetravalent NAb, with partial protection against DENV1 but not against DENV2. Although shuffling technology is a tempting approach when thinking about protection involving the four serotypes, it is uncertain how protein folding can be altered, impacting its antigenic properties.

The vast majority of preclinical DNA vaccines encoding structural and non-structural DENV proteins use immunocompetent BALB/c mice, which do not mimic the full spectrum of the disease as observed in humans but preserve complete responsiveness to vaccination. Mouse models based on immunodeficient mice such as AG129, a type I/II IFN receptor knock-out animal, are also used for evaluation of protection induced by DNA immunization and serotype cross-reactions leading to ADE (118). However, studies with animals lacking IFN responses limit interpretation of some results, especially for DNA vaccines which are good inductors of T cell responses.

### DNA Vaccines Based on the Envelope Protein Alone or Its Domains

Relevant advances have been made regarding DNA vaccines based on the envelope protein alone or its domains, especially domain III (EDIII). Since EDIII interacts with surface host cell receptors for virus internalization, it is the principal focus for developing NAb (119). Although prM acts as a chaperone maintaining correct folding of E protein during virus infection, several research groups have shown satisfactory and protective immune responses with strategies based only on the E full-length, the truncated protein or its domains I/II and/or III (120).

Jimenez and Fonseca cloned 94% of the DENV2 E gene into the vector pCI, but failed to observe detectable humoral and cellular immune responses in mice (88). These undesirable results may be related to the absence of a strong signal peptide for secretion of the recombinant E protein when prM is not present. Supporting this idea, another group constructed a DNA vaccine encoding the NGC DENV2 ectodomain of the envelope protein (domains I, II, and III), corresponding to 80% of the E protein, fused to the signal peptide sequence of the human tissue plasminogen activator (t-PA) (94). The t-PA/E sequence was cloned into pcDNA3 vector generating the pE1D2 vaccine, which mediated secretion of the recombinant protein. BALB/c mice i.m. immunized with pE1D2 presented DENV2 NAb and T-cell responses with IFN-γ production (94, 95). Moreover, all vaccinated animals survived a DENV2 lethal intracerebral challenge. Further studies revealed the importance of T-cell responses in pE1D2-conferred protection, mainly CD4^+^ T lymphocytes, once depletion of these cells completely abolished protection (96). Authors also identified 7 epitopes from E protein that were recognized by CD4^+^ and/or CD8^+^ T cells after pE1D2 immunization and resulted in IFN-γ and TNF-α production. Such results suggest activation of polyfunctional T cells involved in protection against dengue.

The group also constructed a DNA vaccine based only on NGC DENV2 domain III (pE2D2) fused to the t-PA signal sequence (94). However, compared to the ectodomain-based vaccine, pE2D2 was less efficient. BALB/c mice i.m. inoculated with pE2D2 presented only marginal DENV2 NAb levels, though titers of these antibodies increased significantly after virus challenge. Additionally, pE2D2 induced partial protection upon a lethal DENV2 challenge.

Other authors focused on the development of EDIII-based DNA vaccines. Mota et al. (92) cloned EDIII sequences from DENV1 Hawaii strain, DENV2 NGC strain, DENV3 H87 strain and DENV4 H241 strain into pcDNA3 and tested a tetravalent formulation in BALB/c mice. Inoculation of this formulation by the i.m. route elicited significant and homogeneous antibody titers against the four dengue serotypes, but with borderline neutralizing activities. Protection was assessed only in newborn mice inoculated with sera from monovalent- or tetravalent-immunized adult animals and DENV2 NGC virus with observation of 87% survival rate. Nevertheless, the absence of a challenge test with other virus serotypes and the indirect assay based only on NAb for protection evaluation partially compromise the obtained results and does not address the problem of balancing the immune response against all DENV.

Further EDIII-tetravalent approach consisted of synthetic consensus sequences from all DENV serotypes, codon-optimized for expression in human cells and cloned in pVAX1 vector as a single open reading frame (93). The DNA vaccine (pDV-U-DIII) elicited significant tetravalent NAb titers in BALB/c mice inoculated by electroporation, but no *in vivo* protection was investigated. Kulkarni and coworkers also amplified EDIII genes from all serotypes using overlapping primers and ligated them as a single tetravalent gene (98). BALB/c mice i.m. immunized with this DNA vaccine showed tetravalent NAb responses and protection assessed by intracerebral DENV1–4 challenges ranged from 83 to 50%, with the lowest survival against DENV2.

One DNA vaccine based on EDIII was administered by the i.d. route using the gene-gun technology (97). In this case, codon-optimized sequences for expression in mammalian cells, derived from DENV1 Nauru Island, DENV2 NGC, DENV3 3H87, and DENV4 Dominica strains, were fused to the human IgG heavy chain constant domain 3 (γCH3), as an attempt to improve immunogenicity and antigen secretion. BALB/c mice immunized either with monovalent or tetravalent vaccines presented NAb at different levels for each dengue serotype. Immunization with the tetravalent formulation led to antibody titer reduction, indicating imbalancement of the humoral response when the four vaccines were combined. However, authors stated that no antibody cross-reactivity between the different serotypes was observed in serum samples raised after immunization with each monovalent DNA vaccine and tested in THP-1 cells to investigate one possible ADE effect. Another strategy used a DNA vaccine encompassing a single-chain Fv antibody (scFv) sequence, specific for the dendritic cell endocytic receptor DEC205, fused to EDIII sequence from a DENV2 isolated in Brazil. BALB/c mice i.m. inoculated by electroporation with this construct presented strong NAb and specific CD4^+^ T-cell responses (103).

Albeit the great focus on domain III of the envelope protein for inducing neutralizing antibodies, some data demonstrated that domains I/II also contain important epitopes capable of stimulating NAb production in humans (121–123). However, while DIII is coded by a continuous region, genomic sequences of DI and DII are discontinuous and intercalated (11), which poses further difficulties for vaccine constructions based on DI/II alone, concerning correct folding of the recombinant protein and its secretion (124). Additionally, recent studies showed that polyclonal antibodies generated by immunization with a DNA vaccine encoding only the DENV I/II domains enhance other flavivirus infections in the context of ADE, such as Zika, West Nile and Yellow Fever viruses (125).

### DNA Immunization Based on Other Dengue Proteins

Apart from strategies based on the envelope protein and induction of NAb, some candidate vaccines bet on other dengue proteins. One reason is the involvement of prM- and E-directed antibodies in the ADE phenomenon. In addition, there is increasing evidence that both CD4^+^ and CD8^+^ T cells contribute to protection toward DENV, specifically targeting different virus proteins. Recent studies suggest that CD4^+^ T cells are preferentially activated by the E, C, and NS1 proteins while CD8^+^ T lymphocytes are mostly directed to NS3, NS4B, and NS5 (126, 127), although other report showed that anti-DENV3 CD8^+^ T-cell significantly target the structural proteins (128).

The capsid protein is another possible protective antigen. However, no dengue DNA vaccine studies have been reported yet based on this protein, albeit there are studies against other flaviviruses, as West Nile and Duck Tembusu viruses, which induced efficient immune responses and support the inclusion of this antigen in a dengue vaccine (129, 130).

Alternatively, the NS1 is highly immunogenic and is the only virus protein expressed both on the surface of host infected cells and released into circulation, which makes it able to trigger humoral and cellular immunity (24). Actually, most of the antibody response observed in convalescent dengue patients is directed to NS1 and E proteins (131, 132). Anti-NS1 antibodies may recognize the surface anchored protein of infected cells and mediate their lyses by complement-fixing activity and/or cell-mediated cytotoxicity (133, 134) and/or block the pathogenic effect of soluble NS1 (135, 136).

The first report of one NS1-based DNA vaccine was by Wu et al. (137), who constructed one plasmid encoding the full-length NS1 gene from a Taiwanese DENV2, strain PL046, including its signal peptide sequence, corresponding to the C-terminus region of E protein, for secretion of NS1. Immunocompetent C3H mice were immunized with this vaccine (pD2NS1) alone or mixed with other plasmids encoding pIL-2, pIL-4, pIL-12, or pGM-CSF cytokines, and further challenged with a lethal DENV2 dose by the intravenous route. Immunization trigged NS1-specific antibodies and a T-cell proliferative response, and induced high survival rates. Mice inoculated with pD2NS1 together with pIL-12 plasmid were more protected, suggesting the importance of this cytokine in protection mediated by NS1, triggering a Th1 response. The role of anti-NS1 antibodies in protection was also assessed by challenging newborn ICR mice from pD2NS1-immunized dams. Most mice survived virus infection, indicating that anti-NS1 antibodies could pass through the placenta.

A second approach tried to obtain an effective DNA vaccine based on NS1 proteins from DENV and the tick-borne encephalitis virus (TBE), another flavivirus (138). Authors constructed chimeric DNA vaccines encoding fragments of both NS1 proteins but these vaccines failed in conferring protection against the two flaviviruses. Substitution of TBE-derived sequences by DENV-derived sequences has probably disrupted the structure of the protein, altering conformational epitopes involved in the protective immune response.

In contrast, another group presented several evidences of the efficacy of NS1-based DNA vaccines. Initially, four different vaccines were constructed containing the full-length NS1 gene from NGC DENV2 using different signalization sequences (139). The pENS1 vaccine encoded the natural NS1 signal sequence, located at the C-terminal of the E protein, and the NS1 gene, while pcTPANS1 codified the heterologous t-PA secretory signal peptide in frame with NS1 sequence. Both vaccines were designed for driving NS1 secretion. Besides the first upstream signal sequences, two other plasmids (pcENS1ANC and pcTPANS1ANC) encoded the hydrophobic stretch derived from the NS2A protein (ANC) downstream the NS1 gene, to target the protein to host cell surfaces. These constructions differed on their abilities to drive NS1 expression and, consequently, to elicit antibodies. The pcTPANS1ANC plasmid was not efficient for NS1 expression, probably by incompatibilities of the two hydrophobic signal sequences, while the other three vaccines mediated secretion of the recombinant NS1 protein. As expected, only pcENS1ANC led anchoring of the NS1 to plasma membranes, confirming the importance of the ANC sequence for targeting this protein to cell surfaces. BALB/c mice i.m. immunized with pcTPANS1 or pcENS1 vaccines elicited high homogeneous antibody titers, that recognized mainly NS1 conformational epitopes. In contrast, the pcENS1ANC vaccine induced low and heterogeneous antibody response, revealing the negative impact of NS1 anchoring to the cell surface on the antibody response. Furthermore, pcTPANS1 and pcENS1 vaccines were highly protective (97 and 87% survival rates, respectively) after an intracerebral DENV2 lethal challenge. This report corroborated previous evidence of the influence of the recombinant antigen secretion in the efficiency and magnitude of antibody responses elicited by DNA vaccines (140–142). Moreover, the pcTPANS1 vaccine was more protective compared to pcENS1, regarding survival and morbidity rates, demonstrating the efficiency of the t-PA signal sequence to induce a strong immune response in DNA vaccination. Authors also investigated different immunization schemes with the pcTPANS1 vaccine, by administration of two or three DNA doses inoculated by i.m. or i.d. routes. Two i.m. doses were more effective and induced long-term humoral response (143). Anti-NS1 antibodies were detected even 56 weeks after the first plasmid inoculation, with significant antibody titer increase after a booster DNA dose in old animals, which indicated activation of immunological memory. Protection generated by pcTPANS1 was also confirmed by challenging animals with intraperitoneal inoculation of a non-mouse-adapted DENV2 which was isolated from a human patient in Brazil. In this challenge model, DENV2 infected BALB/c mice presented hepatic injuries (micro and macro vacuolization, hepatocyte swelling with cytoplasm rarefaction and altered organelles, and increased levels of serum hepatic enzymes), while in pcTPANS1-vaccinated animals such tissue injuries were absent or significantly reduced (143). Therefore, results revealed the protection efficacy of the pcTPANS1 vaccine against more than one DENV2 genotype.

Contribution of the humoral and cellular immune responses induced by the pcTPANS1 vaccine was further investigated (144). BALB/c mice passively immunized with serum samples collected from vaccinated animals were partially protected by anti-NS1 antibodies, although this protection was abrogated after intracerebral challenge with high DENV2 load. *In vivo* depletion of CD4^+^ lymphocytes completely abolished protection of pcTPANS1-vaccinated mice while 60% of animals survived virus infection after depletion of CD8^+^ cells. Furthermore, adoptive cell transfer of CD4^+^ T cells combined with anti-NS1 antiserum from pcTPANS1-vaccinated mice conferred significant protection in naïve animals challenged with high DENV2 load. Taken together, these results indicated that protection mediated by the pcTPANS1 vaccine required a cooperation between CD4^+^ T cells and the humoral immunity, although the vaccine also activates a CD8^+^ T cells cytotoxic response (144). Authors also investigated whether the pcTPANS1 vaccine induced any hepatotoxicity and no parenchymal or vascular damages in the hepatic tissue or increase in serum levels of liver ALT and AST enzymes were observed after immunization with this DNA vaccine. Therefore, these studies indicated that *in vivo* expression of NS1 mediated by pcTPANS1 was safe, without noticeable pathogenic effects, and provided new insights into the immunity elicited by DENV NS1 and prospects for vaccine development.

Mouse models may be useful tools for studying protection conferred by vaccines and pathogenesis of dengue infections. To complement these studies is essential to identify T-cell epitopes involved in protection and/or pathogenesis, which can also shed light on the responses induced in humans. Hence, due to the importance of T cells in protection generated by the pcTPANS1, authors screened immunodominant epitopes recognized by activated cells after vaccination, using an overlapping peptide library spanning all the NS1 protein (96). Three NS1-derived peptides were able to elicit IFN-γ production in pcTPANS1-immunized mice and four additional peptides were identified after DENV2 challenge. Characterization by intracellular cytokine staining (ICS) revealed that both CD4^+^ and CD8^+^ T cells were involved in IFN-γ and TNF-α production. After virus challenge, authors observed an increase in IFN-γ production but not of TNF-α, which may reflect protective responses. Immunogenicity of those peptides was also investigated in C57BL/6 mice, which have different MHC haplotypes comparing to our BALB/c, and NS1-derived epitopes able to induce IFN-γ responses in both animal strains were identified. Moreover, the identified epitopes may be important for protection not only in mice but also in humans, since some of them were likewise mapped in studies with dengue convalescent patients or volunteers receiving a live attenuated tetravalent vaccine (128). Additionally, other studies with the pcTPANS1 vaccine revealed sustained T-cell response with rapid activation after virus challenge in DENV-infected mice (145).

It's worth to highlight that the NS1 has also been a target for DNA vaccines against Zika virus (ZIKV). This flavivirus spread rapidly in the Americas from 2015 to 2016 and caused serious birth defects in infants whose mothers were infected during pregnancy, including microcephaly and a range of neurological abnormalities and birth defects, termed congenital Zika syndrome (146). Studies demonstrated that NS1-based DNA vaccines, alone or in prime-boost strategy, conferred protection against ZIKV (147–150). One of these studies, using a DNA plasmid that mediated secretion of the ZIKV NS1, showed that vaccine-induced anti-NS1 antibodies may aid in the control of viral replication but are not sufficient to confer protection in the absence of a functional NS1-specific T-cell response (148).

Concerning the other dengue proteins, only few studies have evaluated their use in the context of DNA vaccines. NS3 protein is considered the main target for CD8^+^ T cell responses during dengue infection and the predominance of CTL epitopes suggests its protective role (126, 151). In one report, different DENV2 DNA vaccines were designed based on the NS3 enzymatic domains (152). Authors constructed vaccines encoding the full-length NS3 protein or only the protease or helicase domains, in the presence or absence of the t-PA signal peptide sequence. Protection was evaluated in BALB/c mice i.m. immunized with these constructions and challenged with a lethal dose of DENV2 by the intracerebral route. Results indicated that it depends rather on the NS3 coding region than on the presence or absence of the signal peptide. Most animals immunized with vaccines encoding the full-length NS3 or the helicase domain survived the challenge, although with clinical signs of infection, in particular those immunized with the helicase domain-based DNA vaccines. In contrast, no protection was induced by plasmids encoding only the NS3 protease domain. The vaccines encoding the full-length NS3, regardless the presence of the t-PA signal sequence, elicited a T-cell response revealed by INF-γ production. Thus, the study pointed out that DNA vaccines based on NS3 or helicase domain can in fact be protective against DENV infection, although some limitation was observed when only the helicase domain was used. Combination of NS3 with other DENV proteins can provide more robust protection and be advantageous in DENV vaccine formulations.

One more study evaluated the use of a DNA vaccine based on the DENV NS3 protein, but authors did not analyze protection (153). They investigated the immune response induced in BALB/c mice by a vaccine encoding the DENV3 protease domain and compared it to the response elicited by the NS3 recombinant protein expressed in *E. coli*. T-cell proliferation was observed in cells collected from DNA vaccinated mice, after stimulation with the NS3 protease, but not in cells from animals inoculated with the purified protein. In contrast, anti-NS3 antibodies were detected only in mice immunized with the purified protein. Besides, DNA vaccinated animals produced preferentially IL2 and IFN-γ, while mice immunized with the protein presented mostly IL-4, IL-10, IL-1, IL-6, and TNF-α. Hence, these results emphasized the different immune responses elicited by distinct vaccination strategies with the same antigen.

The NS2B protein, which functions as a cofactor for the NS3 protease, is also able to induce cellular immune responses during viral infection, but there is only one work using its gene in DNA vaccines for antibody production (66). Authors observed that the induced antibodies recognized both native and denatured NS2B protein.

DNA vaccines based on DENV non-structural proteins detailed in this review are summarized in Table 2.

**Table 2:** DNA vaccines based on dengue non-structural proteins.

**Vaccine (study ID)**	**Antigen (serotype)**	**Plasmid vector**	**Strategy (inoculation route)**	**Animal model**	**Immunogenicity**	**Protection**
pD_2_NS1 (137)	NS1 (DENV2)	pcDNA3	Plasmid encoding peptide derived from the C-terminus of E and NS1 (i.m.), alone or mixed with cytokine plasmids: pIL-2, pIL-4, pIL-12, pGM-CSF (i.m.)	C3H and ICR mice	Production of anti-NS1 IgG Detection of antigen-specific T cell proliferation (C3H mice)	82% protection in C3H mice against DENV2 i.v. challenge; Protection against DENV2 s.c. challenge in ICR newborn mice from pD_2_NS1-immunized mother
pTS1042, pTS1057 (138)	NS1 (DENV2 and TBEV)	pMV100	pTS1042 encodes a signal peptide, 62aa from TBE NS1 and last 290 aa from DENV2 NS1 (i.m.) pTS1057 encodes a signal peptide, the first 258 aa of TBE NS1 and the last 290 aa from DENV2 NS1 (i.m.)	BALB/c mice	Antisera from vaccinated and challenged animals recognized TBE NS1	No protection in mice against i.p. challenge with TBEV or i.c. challenge with DENV2
pcTPANS1 (139, 143, 144, 154)	NS1 (DENV2)	pcDNA3	Plasmid encoding t-PA signal peptide and NS1 protein (i.m.)	BALB/c mice	Production of anti-NS1 IgG Positive IFN-γ-ELISPOT responses Protective cooperation between CD4^+^ T cells and Ab	80–97% protection in mice against DENV2 i.c. challenge; Reduction or absent of tissue damages in pcTPANS1-vaccinated mice after i.p. DENV2 challenge
pcENS1, pcENS1ANC (139)	NS1 (DENV2)	pcDNA3	pcENS1 encodes the peptide derived from the C-terminus of E and NS1 (i.m.) pcENS1ANC is similar to pcENS1 plus the N-terminal of NS2A (i.m.)	BALB/c mice	Production of anti-NS1 IgG	87% protection in pcENS1-immunized mice against DENV2 i.c. challenge
pcTPANS3, pcTPANS3H, pcTPANS3P, pcNS3, pcNS3H, pcNS3P (152)	NS3, NS3 protease domain, NS3 helicase domain (DENV2)	pcDNA3	pcNS3, pcNS3H, and pcNS3P encode NS3, helicase domain and protease domain, respectively (i.m.) pcTPANS3, pcTPANS3H, and pcTPANS3P encode t-PA signal peptide fused to NS3, helicase domain and protease domain, respectively (i.m.)	BALB/c mice	Positive IFN-γ-ELISPOT response (full-length NS3 constructions)	70–90% protection in mice inoculated with plasmids containing NS3 or helicase domain against DENV2 i.c. challenge; No protection in mice inoculated with vaccines based on NS3 protease domain
pcDNA3/NS3-DEN3 (153)	NS3 protease domain (DENV3)	pcDNA3	Plasmid encoding NS3 protease domain (i.m.)	BALB/c mice	Detection of NS3-specific proliferation *in vitro* Production of anti-NS3 Ab not detected	Not analyzed
pCAG-NS2B (66)	NS2B (DENV2)	pCAGGSP7	Plasmid encoding NS2B protein (i.m.)	BALB/c	Production of anti-NS2B IgG	Not analyzed

*i.c., intracerebral; i.m., intramuscular route; i.p., intraperitoneal; i.v., intravenous; s.c., subcutaneous; TBE, tick-borne encephalitis virus; PRNT, plaques reduction neutralization test*.

### DNA Vaccines With More Than One Antigen

Several groups used the strategy of developing a dengue vaccine based on two or more viral antigens to obtain broader and more protective immune responses against DENV. An alternative approach is to produce a DNA construct containing immunogenic T-cell epitopes instead of the whole protein, minimizing the influence of the protein structure on the triggered immune response. Chen and coworkers constructed a multi-epitope DNA vaccine in the pcDNA3 vector encoding 15 highly conserved CTL epitopes from DENV1, one located in the E protein and the other 14 derived from non-structural proteins (99). DNA immunization of transgenic mice expressing different human HLA class I molecules elicited significant IFN-γ-producing T-cell responses directed to each of the 15 epitopes, and also secretion of IL-6 or TNF-α directed to some epitopes. Additionally, splenocytes from immunized mice efficiently killed epitope-pulsed splenocytes and DENV1-infected splenic monocytes *in vitro*. Overall, results revealed the ability of this vaccine to elicit specific cellular immune responses, but its protective capacity was only evaluated *in vitro*.

Most groups developing vaccines against dengue containing more than one viral antigen focus on combining of the E and non-structural proteins, in order to stimulate both arms of the immune response, humoral and cellular. Inclusion of the E protein is primarily aimed for inducing NAb, whereas non-structural proteins would stimulate more effective T-cell responses. Vaccine strategies include one construct encoding more than one antigen or the administration of several plasmids, each encoding one viral protein.

The immune response and protective efficacy of a DNA vaccine encoding DENV2 prM/E and NS1 proteins, with or without the GM-CSF adjuvant gene, was investigated in mice (105). Two plasmids encoding DENV1 prM-E-NS1 genes alone (pCAG-DV1/E/NS1) or with GM-CSF gene (pCAG-DV1-GM) were constructed based on pCAGGSP7 vector under the control of a single promoter. The immune response and protection induced by these two vaccines were compared to those generated by a plasmid containing only prM/E genes (pCAG-DV1/E). Immunization of BALB/c mice with pCAG-DV1/E/NS1 or pCAG-DV1-GM resulted in long-term IgG response, with detection of anti-DENV1 antibody up to 10 months after immunization. The pCAG-DV1/E/NS1 vaccine induced the highest anti-DENV1 antibody response. However, immunization with pCAG-DV1/E elicited higher NAb titers than with vaccines encoding NS1. All vaccines induced similar IgG1 and IgG2a levels, suggesting a mix of Th1/Th2 responses. High levels of IFN-γ and IL-2 production were observed upon vaccination with pCAG-DV1/E/NS1 or pCAG-DV1-GM plasmids, and increased levels of IL-10 were detected in pCAG-DV1-GM-immunized mice, which may reflect co-expression of GM-CSF. However, the antibody response and cytokine production, was evaluated only by using DENV1 as antigen, which mirrors responses toward the structural prM and E proteins and not to NS1. All vaccines induced CTL responses but the pCAG-DV1/E/NS1 elicited the highest CTL activity. High survival rates were observed in all vaccinated group after intracerebral DENV1 challenge, though only mice inoculated with vaccines encoding NS1 showed no clinical signs of infection. Thus, this study highlighted the importance of including NS1 in a dengue vaccine composition for induction of more effective immune responses. Moreover, co-expression of prM-E-NS1 with GM-CSF did not show enhancement of the elicited immune response.

Considering the promising results obtained with the DENV1 prM-E-NS1 vaccines, the same group constructed three DENV2 vaccines, containing either prM/E (pCAG-prM/E), prM/E/NS1 (pCAG-prM/E/NS1), or prM/E/NS1 fused to GM-CSF (pCAG-DG) (155). BALB/c mice immunized with pCAG-prM/E presented the highest anti-DENV2 antibody titers, while pCAG-prM/E/NS1 induced more NAbs. These data slightly differed from those observed with the vaccines against DENV1. Besides, immunization with these DENV2 vaccines generated partial protection, with 30, 40, and 60% survival rates after intracerebral DENV2 challenge in mice inoculated with pCAG-prM/E, pCAG-prM/E/NS1, and pCAG-DG, respectively. Although protection rates were much inferior to those observed with DENV1 vaccines, these results agree with the previous study in which vaccines encoding the prM, E, and NS1 proteins were more protective comparing to vaccines codifying only the two structural genes. In addition, in both studies, induction of higher NAb titers did not correlate with greater protection. Indeed, T-cell-based immune responses seem to play an important role in the establishment of protective immunity, corroborating the use of non-structural proteins in the composition of a DNA vaccine against dengue.

Recently, the protective potential of a DNA vaccine composed only of non-structural proteins from DENV2 was evaluated in a murine model (156). Three recombinant plasmids based on the pVAX-1 vector were constructed encoding NS1, NS3, and NS5 from DENV2. C57BL/6 mice were i.m. immunized with each of these vaccines individually or with a combination of the three plasmids. All immunizations induced T-cell responses with IFN-γ-production but no antibody production, probably by the lack of a signal peptide in the DNA constructions. Protection was only analyzed in animals immunized with the mixed vaccines and challenged with DENV2 by the intracerebral route. All animals survived virus infection, although no morbidity rates were evaluated. These results confirm the protective role of DENV non-structural proteins albeit the influence of each protein in this protection was not investigated.

Table 3 summarizes the DNA vaccines combining more than one DENV antigen.

**Table 3:** DNA vaccines with more than one antigen.

**Vaccine (study ID)**	**Antigen (serotype)**	**Plasmid vector**	**Strategy (inoculation route)**	**Animal model**	**Immunogenicity**	**Protection**
pcDNA™ 3.1/myc-His (-) A-DENV-1-Meg (99)	CTL epitopes derived from E, NS2A/2B, NS3, NS4A/4B and NS5 (DENV1)	pcDNA3	Plasmid encoding 15 conserved CTL epitopes derived from DENV1 E, NS2A–NS5 (i.m.)	HLA-A*0201 HLA-A*1101 HLA-A*2402 transgenic mice	Positive peptide-specific IFN-γ-ELISPOT responses	Not analyzed
pCAG-DV1-GM, pCAG-DV1/E/NS1 (157)	prM, E, NS1 (DENV1)	pCAGGSP7	Plasmid pCAG-DV1/E/NS1 encodes prM-E-NS1 (i.m.) Plasmid pCAG-DV1-GM encodes prM-E-NS1 fused to GM-CSF (i.m.)	BALB/c mice	Production of anti-DENV1 IgG Positive PRNT_50_ Positive IFN-γ and IL2-ELISPOT responses DENV1-Specific CTL responses	100% protection in mice against DENV1 i.c. challenge
pCAG-prM/E/NS1 pCAG-DG (157)	prM, E, NS1 (DENV2)	pCAG	Plasmid pCAG-prM/E/NS1 encodes prM-E-NS1/i.m. Plasmid pCAG-DG encodes prM-E-NS1 fused to GM-CSF (i.m.)	BALB/c mice	Production of anti-DENV2 IgG Positive PRNT_50_	40–60% protection in mice against DENV2 i.c. challenge
pNS1, pNS3, pNS5 (156)	NS1, NS3, and NS5 (DENV2)	pVAX1	Plasmid pNS1 encodes NS1 (i.m) Plasmid pNS3 encodes NS3 (i.m.) Plasmid pNS5 encodes NS5 (i.m.)	C57BL/6 mice	Positive IFN-γ-ELISPOT responses (single and mix immunizations) Production of anti-NS1, -NS3, or -NS5 Ab not detected	100% protection in mice inoculated with plasmid mix against DENV2 i.c. challenge

*i.c., intracerebral; i.m., intramuscular route; PRNT, plaques reduction neutralization test*.

### Combining DNA Immunization With Other Vaccine Strategies

Heterologous prime-boost immunization including the DNA vaccine strategy is a promising alternative for enhancement long-term immunogenicity and protective efficacy elicited by dengue vaccines. Induction of both humoral and cellular immunity toward one specific antigen using different delivery systems can direct the immune response to a complementary protective outcome. Apparently, the use of distinct strategies presents the antigen to the immune system in a different way, which may involve more than one presenting cell type, generating better immune responses, both in magnitude and quality.

Among different prime/boost immunization regimens against dengue, the most frequent is the combination of DNA and purified recombinant proteins. Protein-based vaccines typically elicit predominant humoral immune responses, while DNA vaccines are good T-cell immunity inducers and, to a lesser extent, antibody production.

The first heterologous prime/boost strategy for dengue with a DNA vaccine was reported as an attempt to improve short-term immunogenicity of a recombinant DENV2 E protein fused to maltose-binding protein (E-MBP) from *E. coli* (158) and a DENV2 DNA plasmid containing prM/E genes (p1012D2ME), previously described in this review (104). The study was conducted in BALB/c mice and immunization regimens consisted of primming with the DNA vaccine or protein followed by two booster doses with the other vaccine, three doses of both vaccines given simultaneously, or each vaccine given individually. The E-MBP protein alone, p1012D2ME-prime/protein-boost or simultaneous immunization with both vaccines elicited higher NAb titers compared to administration of the DNA vaccine alone or protein-prime/DNA-boost strategy (159).

The same group also assessed immunogenicity of these two strategies together with an inactivated DENV2 vaccine (160). The follow-up work was conducted in rhesus monkeys with heterologous prime/boost using DNA, recombinant E protein, and inactivated virus vaccine, or simultaneous DNA/protein and DNA/virus administration. Antibody response was observed in all groups, with co-delivery of DNA/protein resulting in the highest NAb titers. However, only monkeys immunized with inactivated virus vaccine became protected against subcutaneous DENV2 challenge and results discouraged the use of such strategy for a dengue vaccine. Nevertheless, in this work authors immunized animals by i.m. injection and recent studies with DNA administration by electroporation revealed significant improvement of the induced immune response, which could be an alternative for better results.

Another NHP preclinical study was reported with simultaneous immunization of a DNA vaccine encoding the DENV2 EDIII region, one recombinant protein composed of EDIII copies coupled with multimeric E2 proteins from *Geobacillus stearothermophilus*, and one plasmid encoding IL-12 gene (161). Animals presented significant DENV2 NAb and became protected against DENV2 challenge. No animal presented viremia in FRNT50 infectious assay, although virus genome was detected in 3/6 monkeys in RT-PCR analysis (161).

Zang and coworkers evaluated the effect of a bivalent DNA-protein combination using EDIII from DENV1 and DENV2 (162). They inoculated BALB/c mice with the recombinant bivalent protein pro-D1/D2EDIII produced in *E. coli* and the DNA vaccine pcDNA-D1/2EDIII in prime/boost regimen, or the two vaccines administered simultaneously by different routes. Combination of DNA and protein vaccines induced significantly higher NAb titers against both DENV1 and DENV2 compared to single administrations. Konishi et al. (90) also observed that simultaneous administration of subviral extracellular particles and a prM/E-derived DNA vaccine induced higher NAb titers in mice than those provided by immunization with each immunogen isolatedly. All these results corroborated the idea that combination of DNA vaccines with recombinant proteins is highly efficient for inducing NAb, especially by simultaneous co-immunization.

The vast majority of studies evaluating prime-boost strategies using DNA vaccine platforms are based on prM/E proteins as antigens. As far as we know, there are only two reports that analyzed this approach including the NS1 antigen. Mellado-Sánchez and colleagues constructed DNA vaccines containing the full-length envelope (pcDNA 3.1/E) and NS1 (pcDNA 3.1/NS1) genes from DENV2 and combined them to purified E and NS1 recombinant proteins expressed in *E. coli* (GST-E and GST-NS1) (163). BALB/c mice were immunized with three doses of both DNA vaccines, followed by boosting with GST-E and GST-NS1 proteins. Authors observed a significant increase in antibody response to NS1 and in NAb titers when compared to results obtained by isolated inoculation with the DNA vaccines or proteins. The same group constructed another DNA vaccine encoding part of II and III domains of the E protein and NS1 (pEII^*^EIII/NS1^*^) and tested it with GST-E and GST-NS1 proteins (164). BALB/c mice were primed with the DNA vaccine and subsequently boosted with recombinant proteins, but not all animals presented anti-E and anti-NS1 antibodies and only 40% of them exhibited NAb.

The first dengue tetravalent vaccine employing heterologous prime/boost strategy was conducted in rhesus monkeys primed with the tetravalent prM/E-based DNA vaccines (TVDV) or the tetravalent alum-adjuvanted purified inactivated virus (TPIV) vaccine followed by tetravalent live-attenuated virus (TLAV) immunization (165). The tetravalent DNA vaccine was tested only in the first experiment when authors immunized monkeys with two doses of TVDV or TPIV and increased the immune responses by a single TLAV inoculation 2 months later. Results showed positive tetravalent seroconversion with homogeneous NAb titers among immunized groups. Animals primed with the tetravalent DNA vaccine showed reduced viremia compared to control animals, while no viremia was detected in TPIV/TLAV-immunized group after a DENV3 challenge. It is important to note that, although not completely free from viremia, the highest anamnestic immune response was detected in the group inoculated with TDNA/TLAV combination. Possibly, variations in prime/boost regimens with DNA vaccines and inoculation by electroporation could induce more protective and long-lasting immunity. Results from the first randomized Phase-1 clinical trial with heterologous TPIV/TLAV prime/boost vaccine combination in 80 volunteers have been published recently (166).

One pVAX1-derived plasmid (pV-cE80) containing a consensus sequence of the DENV E ectodomain was evaluated in mice together with cE80 purified protein (167). The consensus cE80 sequence has 87–94% nucleotide homology between E ectodomains of DENV1–4 serotypes. Three doses of pV-cE80 were injected alone or in DNA/protein protocols. Heterologous DNA/DNA/protein regimen led to seroconversion with high NAb and increased production of IFN-γ and IL-4 directed to the four DENV serotypes. Three weeks after immunization, vaccine-induced protection was evaluated in a tetravalent intracerebral challenge. DENV2 and DENV4 survival rates of 100 and 62%, respectively, were recorded in DNA/DNA/protein-immunized mice. Protection against DENV1 and 3 was reported as less expressive body weight loss compared to the control group.

Another approach used prime-boost schedules combining DNA vaccine with non-replicative or replicative live vectors, such as vaccinia virus, adenovirus, or yellow fever vaccine. Combination of these vaccine platforms generally aims to improve cellular immune responses. Chen and coworkers evaluated heterologous prime-boost protocols using one DENV1 DNA vaccine and virus replicon particle (VRP) containing full-length prM and E genes (168). This DNA vaccine (D1ME-DNA) was previously tested in NHP with only partial protection (81, 169). The VRP was a replication-deficient recombinant viral vector based on Venezuelan equine encephalitis (VEE) virus, which is efficient in infecting dendritic cells. Cynomolgus monkeys were immunized by three different schemes: DNA-DNA-DNA, VPR-VPR-VPR, and DNA-DNA-VPR. All animals seroconverted but the group receiving DNA-prime/VRP-boost presented the highest DENV1 NAb titers. Moreover, only vaccine schedules including DNA immunization elicited T-cell responses. All vaccination regimens generated protection against DENV1, but only DNA-prime/VRP-boost group presented no viremia after virus challenge. Once more, results emphasized the ability of DNA vaccines in inducing T-cell responses and demonstrated its efficiency in heterologous DNA-prime/VRP-boost regimen eliciting.

Using a similar strategy, Khanam and coworkers constructed one bivalent DNA vaccine encoding a chimeric DENV2/4 domain III from E protein (pVAX-EDIII-4/2) and Adenovirus 5 vector encoding the same bivalent antigen fused to the green fluorescent protein gene (GFP) tag (rAd-Bg) (170). BALB/c mice primed with recombinant adenovirus and boosted with pVAX-EDIII-4/2 produced antibodies that recognized both EDIII and neutralized DENV2 and DENV4. Significant *in vitro* proliferative response with INF-γ secretion was also observed. Unfortunately, authors did not investigate *in vivo* protection.

In another study, George and Eo evaluated prime-boost regimens combining DNA vaccine, replication-deficient adenovirus, and vaccinia virus expressing DENV2 E protein (169). Priming with recombinant vaccinia and boosting with adenovirus rAd-E induced the highest anti-E IgG levels, but titers rapidly decreased. Comparison of cellular immune responses revealed different CD4^+^ and CD8^+^ T cell activation patterns. Priming with vaccinia virus induced stronger CD8^+^ while with adenovirus elicited robust CD4^+^ T cell responses, and booster with the DNA vaccine significantly enhanced both responses. Once more, assessing the protective capacity of these approaches would help to predict more effective vaccine schedules.

One chimeric yellow fever (YF)/dengue 2 virus, based on the backbone of the live attenuated YF vaccine (YF17D), was also evaluated in combination with the DNA vaccine pE1D2 (95). The pE1D2 (94) encodes the ectodomain of the DENV2 E protein fused to the t-PA signal peptide, while the chimeric YF/DENV2 (YF17D-D2) was constructed by replacing the prM and E genes from YF17D with those of DENV2 genes (171). This YF17D-D2 is similar to one of the chimeric viruses that composes Dengvaxia (53). BALB/c mice were immunized with different protocols: pE1D2-prime/YF17D-D2-boost; YF17D-D2-prime/pE1D2-boost; the mixture of both vaccines in the same formulation; or simultaneous administration of the two vaccines by different inoculation routes. In all immunization regimens, the combination of the two vaccines generated a synergistic effect on the antibody response by increasing NAb titers against DENV2 when compared to levels detected after inoculation of only one vaccine strategy. Moreover, results revealed that the cellular immune response with INF-γ production was induced mainly by the pE1D2 DNA vaccine. These data not only corroborate other studies showing the strong induction of T-cell responses by DNA vaccines but demonstrated, as well, a poor cellular immune response elicited by the YF17D-D2 vaccine. Thus, it also shed light on why the Dengvaxia, which is composed of four chimeric YF/dengue viruses similar to YF17D-D2, was not as protective as expected, despite the induction of NAb against all dengue serotypes. Moreover, combination of pE1D2 and YF17D-D2 vaccines generated complete protection in BALB/c mice after challenge with DENV2, with 100% survival and no morbidity. Simultaneous administration of pE1D2 and chimeric virus YF17D-D2 generating complete protection is an attractive option to avoid long immunization periods which may represent one problem in dengue-endemic regions. In fact, the commercial YF/DENV Dengvaxia requires administration of three doses given 6 months apart to achieve satisfactory NAb titers against all serotypes, which can leave the population more susceptible to the development of severe disease until it became immunologically covered and protected. Co-administration of DNA vaccines and chimeric YF/dengue may circumvent such difficulty by reducing the number of required doses and shortening immunization time.

Taken together, these works offer innovative ideas regarding the development of dengue vaccines administered in a prime/boost regimen or by simultaneous immunizations, using new vaccine candidates, or by strengthening immunity induced by vaccines that are already in clinical trials.

The DNA vaccine strategies combined with other vaccine platforms are listed in Table 4.

**Table 4:** Heterologous prime/boost strategies in dengue DNA vaccines.

**Vaccine (study ID)**	**Antigen (serotype)**	**Plasmid vector**	**Strategy (inoculation route)**	**Regimen**	**Animal model**	**Immunogenicity**	**Protection**
P1012D2ME + DEN-2 MBP (159)	prM and E (DENV2)	pVR1012	Plasmid p1012D2ME encodes prM-E (i.d.) + DEN-2 MBP (EDIII-MBP fusion protein expressed in *E. coli*) (i.m.)	D/P/P P/D/D D+P/D+P/D+P	BALB/c mice	Positive PRNT_50_ Simultaneous administration with DNA + protein induced slightly higher NAb titers	Not analyzed
DENV2 DNA vaccine + inactivated DENV2 vaccine + DEN-2 MBP (160)	prM and E (DENV2)	proprietary plasmid vector	DENV2 DNA vaccine (i.d.) + Purified inactivated DENV2 vaccine (PIV) (i.m.) + DEN-2MBP (fusion protein expressed in *E. coli*) (i.m.)	D/P/P D/IV/IV D+P/D+P/D+P D+IV/D+IV/D+IV	Rhesus monkeys	Positive PRNT_50_ Inactivated DENV vaccine induced the highest anti-DENV2 and anti-NS1 Ab titers Simultaneous delivery of DNA + protein induced the highest NAb titers	Only inactivated DENV vaccine induced protection in monkeys against s.c. DENV2 challenge
EDIII-E2 (161)	EDIII (DENV2)	pEMC	Plasmid encoding EDIII (i.d.) + EDIII coupled on scaffold from *Geobacillus stearothermopilus* E2, fusion protein expressed in *E. coli*) (i.m.) + IL-12 plasmid (i.d.)	P+D+ IL-12 DNA/P+D+ IL-12 DNA/P+D+ IL-12 DNA	Rhesus monkeys	Production of anti-EDIII Ab Positive FRNT_50_ against DENV2 Negative PRNT_50_ against heterologous serotypes	All vaccinated monkeys protected against i.m. DENV2 challenge (no viremia detected by infectious assay), but DENV was detected by RT-PCR in 3/6 vaccinated animals
pcD2ME + P2EP (90)	prM and E (DENV2)	pcDNA3	Plasmid pcD2ME encoding prM-E proteins (i.m.) P2EP (prM-E proteins expressed in mammalian cells) (s.c.)	D+P/D+P	ICR mice and ddY mice	Positive PRNT_90_ Simultaneous delivery of DNA + protein induced higher NAb titers than single immunization (pcD2ME or P2EP)	Not analyzed
pcDNA-D1/2EDIII + recombinant pro-D1/D2EDIII protein (162)	EDIII (DENV1 and 2)	pcDNA3	Plasmid pcDNA-D1/2EDIII encodes EDIII of DENV1 and 2 (i.m.) + Pro-D1/D2 EDIII protein (EDIII of DENV1 and 2 expressed in *E. coli*) (i.p.)	D+P/D+P/D+P	BALB/c mice	Positive PRNT_50_ against DENV1 and DENV2Combination of DNA and protein induced higher NAb titers than vaccines alone	Not analyzed
TDNA TPIV TLAV (165)	prM and E (DENV1–4)	pVR1012	TDNA- plasmids encoding DENV1–4 prM-E (i.m.) + TPIV—tetravalent purified inactivated DENV vaccine (PIV) (i.m.) + TLAV—tetravalent attenuated DENV vaccine (s.c.)	D/D/AV IV/AV AV/AV	Rhesus monkeys	Positive PRNT_50_ against DENV1–4	Reduction of viremia in group primed with DNA vaccine and s.c. DENV3 challenged; Full protection (no viremia) in IV/AV and group against DENV1–4 challenge
D1ME-DNA + D1ME-VRP (168)	prM and E (DENV1)	pVR1012	Plasmid D1ME-DNA encodes prM-E (i.m.) + D1ME-VRP encodes prM-E (i.m.)	D/D/VRP	Cynomolgus macaques	Positive IFN-γ-ELISPOT Production of anti-DENV1 Ab Positive PRNT_50_ Prime-boost induced the highest IgG and NAb titers. Only vaccine schemes that used DNA vaccines were able to induce T cell response	100% protection in prime-boost vaccinated cynomolgus macaques and challenged by DENV1 subcutaneous injection (no viremia)
pVAX-EDIII-4/2 + rAd-Bg (170)	EDIII (DENV2 and 4)	pVAX1	Plasmid pVAX-EDIII-4/2 encodes EDIII of DENV2 and DENV4 (i.d.) + Adenovirus rAd-Bg encodes EDIII of DENV2 and DENV4 fused to GFP (i.p.)	Ad/D/D/D	BALB/c mice	Production of anti-EDIII antibodies (DENV2 and 4) Positive PRNT_50_ against DENV2 and DENV4	Not analyzed
pDE ± rAd-E ± VV-E (169)	Envelope (DENV2)	pCI-neo	Plasmid pDE encodes E (i.m.) + Adenovirus rAd-E encodes E (i.m.) + Vaccinia virus VV-E encodes envelope (i.m.)	D/Ad, D/VV, Ad/D, Ad/VV, VV/D, VV/Ad	BALB/c mice	Prime with VV induced a strong E-specific CD8^+^ T cell responses while prime with Ad elicited a strong E- specific CD4^+^ T cell responses; DNA-boost increased both T cell response	Not analyzed
pE1D2 + YF17D-D2 (95)	Ectodomain of E (DENV2)	pcDNA3	Plasmid pE1D2 encodes ectodomain of E protein (i.m.) + Virus YF17D-D2 encodes prM-E (s.c) or Mix (pE1D2+YF17D-D2, i.m.)	D+AV D/D/AV AV/D/D D+AV/D+AV Mix Mix/Mix	BALB/c mice	Positive IFN-γ-ELISPOT Positive PRNT_50_; Prime-boost (3 doses) or vaccine combination (2 doses) induced the highest titers of NAb; Cellular immune response was induced predominantly by DNA vaccine.	100% survival and 0% morbidity rates in mice against DENV2 i.c. challenge
pcDNA 3.1/E and pcDNA 3.1/NS1 + GST-E and GST-NS1 (163)	E and NS1 (DENV2)	pcDNA3	plasmid pcDNA 3.1/E encodes envelope (i.m.) + plasmid pcDNA 3.1/NS1 encodes NS1 (i.m.) + GST-E and GST-NS1 fusion proteins expressed in *E. coli* (i.d.)	D/D/D/P	BALB/c mice	Production of anti-E and anti-NS1 Ab Positive PRNT_50_ DENV2 Prime-boost induced higher levels of anti-NS1 Ab and Nab comparing to DNA vaccine alone	Not analyzed
pEII*EIII/NS1*+ GST-E and GST-NS1 (164)	E and NS1 (DENV2)	pcDNA3	Plasmid pEII*EIII/NS1* Encodes domains II/III of E and NS1 (57-131aa) (i.m.) + GST-E and GST-NS1 fusion proteins expressed in *E. coli* (i.d.)	D/D/D/P	BALB/c mice	Production of anti-E and anti-NS1 antibodies Low level of Nab	Not analyzed

*GFP, green fluorescent protein; GST, Glutathione S-transferase; MBP, maltose-binding protein; i.c., intracerebral; i.d., intradermic route; i.m., intramuscular route; i.p., intraperitoneal route; s.c., subcutaneous route; EDIII, domain III of envelope protein; NAb, Neutralizing antibody; PRNT, plaques reduction neutralization test; FRNT, focus reduction neutralization test; Ad, Adenovirus; VV, vaccinia virus; Virus YF17D, attenuated yellow fever 17D virus; PIV, purified inactivated virus; TPIV, tetravalent purified inactivated virus; TLAV, tetravalent live-attenuated virus. In the regimen column: + indicates that vaccines were simultaneously administered; / indicates the interval between immunizations; D, DNA vaccine; P, protein; IV, inactivated virus; AV, attenuated virus; Ad, Adenovirus; VV, Vaccinia virus; VRP, virus replicon particle*.

## Discussion

In this paper we reviewed studies with DNA vaccines against DENV. We also presented dengue vaccines that have been tested in the past few years in clinical trials to point out difficulties of all the different approaches in attaining robust and protective immune responses against the four serotypes. Development of an effective vaccine against DENV is not an easy task, mainly because of a tetravalent vaccine necessity, without risk of induction severe dengue in subsequent infections in vaccinated individuals not completed protected. Hence, the greatest problem is that an inefficient DENV vaccine can be worse than non-immunization, due to the complexity of the disease and the involvement of strong but not protective immune responses in DHF/DSS.

Another point that also impacts the development of dengue vaccines is the strong cross-reaction of antibodies and T-cell responses between DENV and the Zika virus (172). Infection with ZIKV, a flavivirus with high similarities to DENV, was considered of minor importance before the virus reach Brazil and rapidly spread throughout Latin America. After 2015 several investigations revealed that ZIKV infection in pregnant women may have serious consequences to the fetus, causing abortion or baby births with congenital Zika syndrome (146). The role of immune response cross-reactions between DENV and ZIKV in protection or exacerbation of the pathogenesis of both diseases is still not fully understood. Some reports suggest that antibodies against DENV can cause ADE in subsequent infection with ZIKV and vice-versa (173, 174), while other studies indicate cross-protection (172). Consequently, nowadays it is desirable that dengue vaccines are no longer tetravalent but pentavalent, including Zika antigens. It is worth noting that after the huge Zika epidemics in the Americas, DNA vaccines were the first tested against this virus in experimental animals as well as in human trials (175–177).

The fact that DNA vaccines are able to elicit humoral and cellular immune responses makes them an attractive approach to control these viruses. More recently, T-cell responses have drawn attention of scientists as several reports demonstrated their participation in achievement of protective and robust dengue immunity. Moreover, the presence of multifunctional T cells producing different cytokines at the same time and sometimes with cytotoxic profiles, detected in convalescent dengue patients and also in clinical trials with volunteers immunized with live attenuated virus vaccines (51, 52), emphasize the role of T cells in attaining broad and long-term protection against the different serotypes. We believe that robust protection against all DENVs should activate antibody and T cell responses.

Initial results in clinical trials with DNA vaccines were not as efficient as those observed with experimental animals, especially because of the low transfection rates in humans. However, these obstacles have been overcome by using different immunization methods, such as electroporation, and/or immunostimulatory sequences like CpG ODN and GM-CSF adjuvant factors (67). One important point is the targeting of the vaccine to children, since they are one of the most dengue vulnerable population. Although little data on pediatric clinical studies with DNA vaccines are available, reports conducted with therapeutic DNA vaccine in HIV-infected children (178) and in prime/boost regimen against influenza (179) revealed that DNA immunization was well tolerated and safe.

Another relevant element concerning dengue DNA vaccines is the possibility to use them combining with other strategies. In fact, T-cell responses elicited by DNA vaccines seem to be more robust. One study combining a DNA vaccine based on the ectodomain of the DENV envelope protein and a chimeric YF/DENV attenuated virus revealed increased NAb and T-cell responses comparing to both strategies used separately (95). Such combination induced synergistic effect on the humoral response, while the cellular immune response with IFN-γ production was driven mostly by the DNA vaccine. Other investigations with heterologous prime/boost protocols using DNA vaccines revealed, likewise, a substantial increase of cellular immune responses (180). Combination of different dengue antigens may also be an interesting approach since the immune responses toward them can prevent and limit viral infection in distinct moments. This includes vaccines based on structural and non-structural proteins against all dengue serotypes, and none of the vaccines currently in clinical trials presents NS from the four DENV.

In summary, DNA vaccines are a powerful tool for obtaining more effective dengue and other flavivirus vaccines, both in simple formulations or combining with other strategies. We believe that heterologous prime-boost immunizations using DNA as one of the vaccine approaches may overcome problems observed with the licensed DENV vaccines or those presently in phase 3 trials. New data should emerge in the near future-proofing the efficiency of DNA vaccines, which will achieve, in fact, the third vaccine generation status for its clinical use.

## Author Contributions

AA conceived the manuscript. AA, PP, and SC wrote the paper. All authors contributed to the article and approved the submitted version.

## Conflict of Interest

The authors declare that the research was conducted in the absence of any commercial or financial relationships that could be construed as a potential conflict of interest.
